# Editorial: Families and Functioning in Childhood and Adolescence

**DOI:** 10.3389/fresc.2022.858239

**Published:** 2022-03-24

**Authors:** Liane Simon, Olaf Kraus de Camargo

**Affiliations:** ^1^Department of Family, Child and Social Work, MSH Medical School Hamburg, Hamburg, Germany; ^2^Department of Pediatrics, McMaster University, Hamilton, ON, Canada

**Keywords:** editorial, introduction, families, childhood, adolescence, functioning, authors

In WHO's International Classification of Functioning, Disability, and Health (ICF), “functioning” refers to the sum of functions and structures of the body and mind, the actions people perform, and the execution of activities when participating in diverse life situations. These interact with personal and environmental factors. Functioning in childhood and youth is highly dependent on the family. Every family is different, and the concept of family has changed over time and is diverse in different geographic regions of the world. According to the American Academy of Family Physicians: “A family is a group of individuals with a continuing legal, genetic, and/or emotional relationship. Society relies on the family group to provide for the economic and protective needs of individuals, especially those who are vulnerable.” (1).

In the field of child development disciplines (e.g., early intervention, developmental paediatrics, social paediatrics, paediatric rehabilitation, child and youth mental health, etc.) the so-called “main complaints” are brought to the attention of professionals by family members or caregivers. We see and support families who live and struggle with their children and youth. Supporting and encouraging the people with whom a child lives and interacts and improving other environmental factors promotes a child's activity and participation opportunities and improves the child's quality of life. It is a person-centred approach taking into consideration a child's life situation. On the other hand, we know that goals defined by parents would not necessarily match with those a child would describe as meaningful for them (2).

Authors from around the world followed our invitation to publish their ideas and research findings about how to engage and consider family aspects in working with children and adolescents. Those ideas share a common approach in seeing a person within their environment and life situation (3). We are excited to share an extremely diverse, intriguing, and thought-provoking collection of contributions. Embracing a family-centred attitude, some of the articles were co-written with families (parents and youth). This intimate collaboration reflects a paradigm shift described by Nowotny et al. (4) in their book “Re-thinking Science”: instead of science speaking to society, we are living in a time where society speaks to science. New knowledge and insights are co-created and developed within meaningful contexts. The science ivory tower is open to the agora of the people for an inclusive conversation.

“Scientists listening to families” is the topic of two articles that describe the perspectives of siblings and fathers, two groups often easily overlooked when caring for children with complex or chronic health conditions (Nguyen et al.; Ogourtsova et al.). Another easily overlooked aspect is that of non-traditional family constellations such as LGBTQ + parents and polygamous and polyamorous families. Phoenix et al. describe how clinicians and service providers can easily create barriers for them to access services by perpetrating systemic patterns of racism, sexism, and ableism. In this vein also belongs the reflection of Reitzel et al. on how an intersectional lens between personal and environmental factors can help us identify the risk for discrimination by expanding the use of the ICF.

With relation to service structures, the importance of family-centred care and how it influences functioning of children and adolescents is described by Rosenbaum who also contributes extensively in disseminating those ideas around the world, as the example from Brazil in this collection demonstrates (Airoldi et al.).

One of the important contributors to child and adolescent functioning is the perceived parental social support as described by Weiss et al. following families with autistic children longitudinally. Such support is further compromised the current COVID-19 pandemic for families with children with disabilities impacting their quality of life as shown by Ali et al. with data from Pakistan.

When families are not available, and children or youth are being cared for by child protection services, different aspects and factors need to be considered. Kim et al. demonstrate in their review of longitudinal studies through the “ICF lens” how having a distal and proximal assessment of functioning can help in understanding the different risks and trajectories observed in children in care. This insight is innovative and might help in conceptualizing future longitudinal studies within the ICF framework.

We hope that these ideas stimulate our readers to reflect on their own practice and help them to develop inclusive approaches to engage children and families in clinical practice and research.

## Author Contributions

All authors listed have made a substantial, direct, and intellectual contribution to the work and approved it for publication.

## Conflict of Interest

The authors declare that the research was conducted in the absence of any commercial or financial relationships that could be construed as a potential conflict of interest.

## Publisher's Note

All claims expressed in this article are solely those of the authors and do not necessarily represent those of their affiliated organizations, or those of the publisher, the editors and the reviewers. Any product that may be evaluated in this article, or claim that may be made by its manufacturer, is not guaranteed or endorsed by the publisher.
